# CT Appearances of Ketamine-Induced Uropathy: A Guide to General Radiologists

**DOI:** 10.7759/cureus.111854

**Published:** 2026-06-30

**Authors:** Gayathri Yapa, Hamza Khan

**Affiliations:** 1 Radiology, Queen Elizabeth Hospital, Lewisham and Greenwich NHS Trust, London, GBR; 2 Urology, Queen Elizabeth Hospital, Lewisham and Greenwich NHS Trust, London, GBR

**Keywords:** computed tomography urogram, imaging, ketamine, radiologist, uropathy

## Abstract

Ketamine-induced uropathy (KIU) is a chronic inflammatory condition in the urinary tract, particularly involving the urinary bladder, in patients with chronic ketamine abuse. It is a growing clinical issue in the younger population, affecting the quality of life. A 28-year-old female patient presented to the Accident and Emergency Department with on-and-off flank pain for one week and later developed severe left flank pain. She also had long-standing urine incontinence with a five-year history of ketamine abuse. A non- contrast computed tomography of the kidneys, ureters, and bladder (CT KUB) was performed to rule out urinary tract stones in the acute clinical presentation. Later, a multiphase CT urogram was performed to assess the renal tracts and urinary bladder on suspicion of KIU. On imaging, a small-volume, thick-walled bladder, perivesical fat stranding, and thickening of the left distal ureter resulting in upstream urinary tract dilatation were observed. Diagnosis of KIU was made by linking the clinical history and the imaging appearances. She was transferred to a specialised centre for management with continuous follow-ups and monitoring of renal and liver functions. The imaging features of KIU are well described in the literature. Typical findings include a contracted small-capacity bladder with perivesical fat stranding, upper urinary tract dilatation, and ureteric wall thickening. However, these features are not specific and may overlap with a range of inflammatory and infective pathologies affecting the urinary tract, highlighting the importance of correlating imaging findings with clinical history and risk factors. Radiologists play a central role in detecting these abnormalities in the acute setting, frequently providing the first indication of an underlying ketamine-related disorder, as ketamine misuse is often not disclosed or recognized at initial presentation. Early recognition of these imaging features can expedite diagnostic workup and support targeted clinical management. The mainstay of treatment is termination of ketamine use, and a multidisciplinary approach is needed for management.

## Introduction

Ketamine is a rapid-onset, short-acting medication primarily used for anaesthesia in the medical field since 1960 due to its dissociative properties [1,2]. It is also used for pain relief and treatment-resistant depression nowadays [2]. Misuse of ketamine is on the rise globally, especially in the younger population (16-24 years), as it is known to have feelings of detachment, stimulation, and euphoria [2,3]. In general, much is still unknown about the epidemiology of ketamine, specifically regarding the frequency of its use and in what settings, and the trends over the years [3]. It is commonly taken as nasal inhalation (snorting), which causes more symptoms than oral use, and is used particularly in night club settings [4,5]. Ketamine is metabolised into norketamine, which is slowly eliminated in urine and bile [2]. This results in cytotoxic and inflammatory effects in the urinary and biliary system [2]. The urinary bladder is the commonest organ affected, which is known as ketamine cystitis.

The imaging features of chronic ketamine-induced uropathy (KIU) are well established; however, a history of ketamine misuse may not be provided on the imaging referral form, making diagnosis challenging. In such cases, the radiologist may be the first clinician to suspect the condition based on characteristic imaging findings. As these features can overlap with those of infection and other inflammatory conditions in the urinary tract, awareness of the typical CT appearances is essential. This case highlights the importance of recognising KIU in the setting of limited clinical information and emphasises the key role of radiologists in facilitating timely diagnosis, leading to appropriate management.

## Case presentation

A 28-year-old female patient presented with on-and-off right flank pain for a week. Later, the pain developed in the left flank associated with nausea and vomiting, without dysuria. She had long-standing urinary incontinence, and the cause was uncertain. No change in her bowel habits, and her periods were regular. Urine dipstick was positive for blood. The clinical impression was acute renal colic, and a non-contrast computed tomography of the kidneys, ureters, and bladder (CT KUB) was arranged.

CT KUB revealed a small-volume urinary bladder measuring 46 mL [6], with left-sided hydroureteronephrosis (Figure 1). No ureteric calculi were seen. Following this, the patient was referred to the Urology Department for further evaluation, and a five-year history of ketamine abuse was identified in her clinical history.

**Figure 1:**
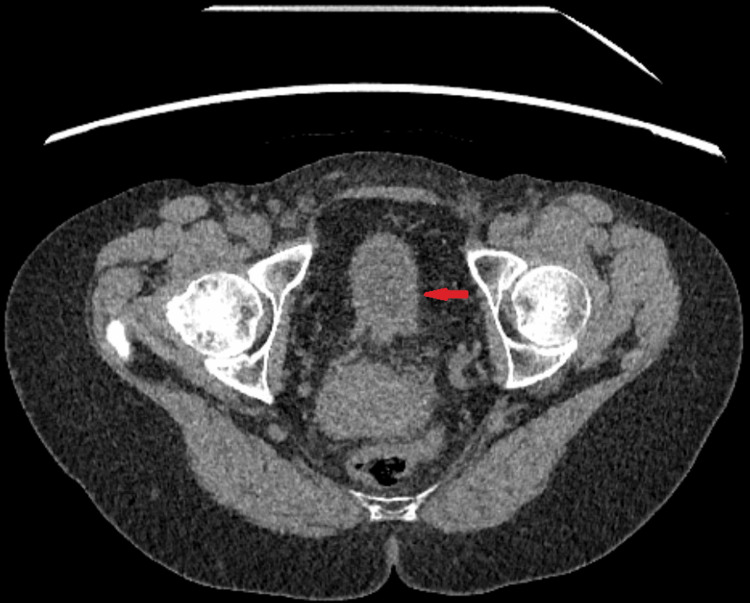
Axial image of the non-contrast CT KUB, showing a small-volume urinary bladder (arrow) (mA = 79, kV = 120) CT KUB: computed tomography of the kidneys, ureters, and bladder

A multiphase CT urogram was performed for better evaluation of the renal tracts following a non-contrast CT KUB on the same day. The imaging protocol included a nephrographic phase (90 seconds following IV contrast injection of OPTIRAY 350 (ioversol), 70 mL) and a delayed phase (15 minutes following IV injection), with a 314 mA and 100 kV. Post-contrast images also confirmed the small volume thick-walled bladder (wall thickness: 6.4 mm). Left distal ureteric wall thickening with a length of 19 mm was demonstrated in the nephrographic phase, with a thickness of 4 mm (Figures 2-3).

**Figure 2:**
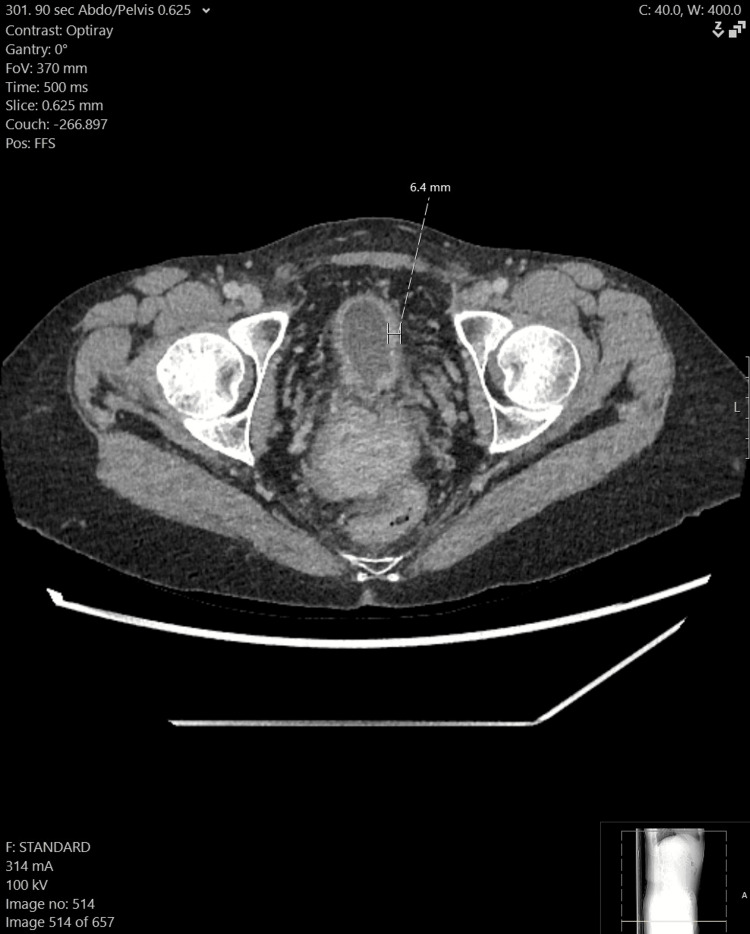
Axial image of the nephrographic phase of the CT urogram, showing a small-volume bladder with a thickened bladder wall

**Figure 3:**
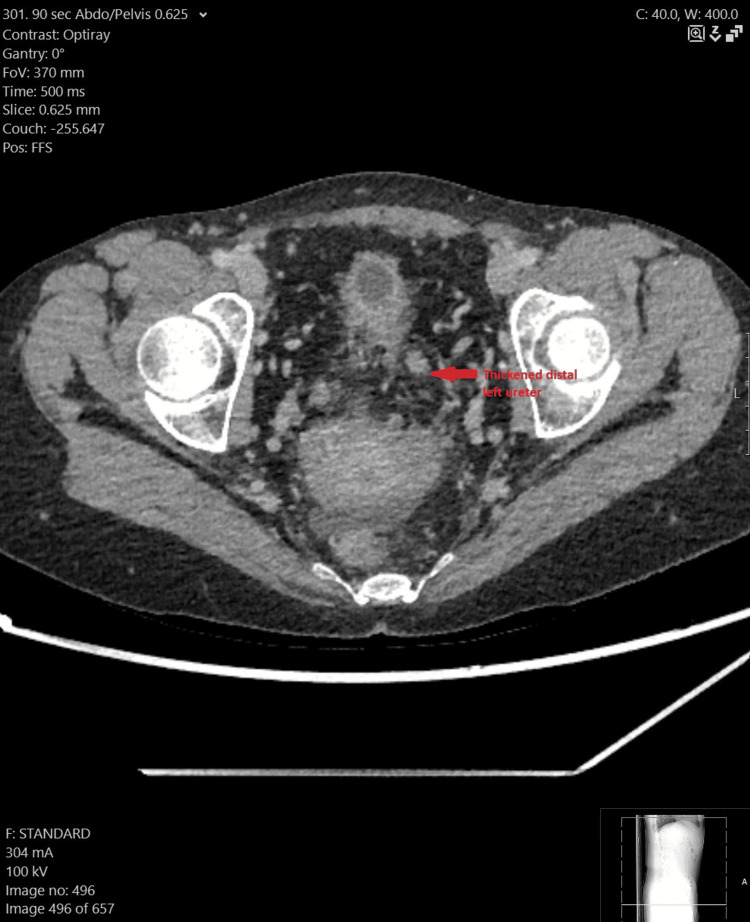
Axial image of the nephrographic phase of the CT urogram showing a thickened left distal ureter (arrow)

There was upstream ureteric dilatation (diameter of proximal ureter was 12 mm) and pelvicalyceal dilatation (AP diameter of renal pelvis was 20.4 mm), with thinning of the cortex (cortical thickness: 4.4 mm) in the nephrographic phase (Figures 4-5).

**Figure 4:**
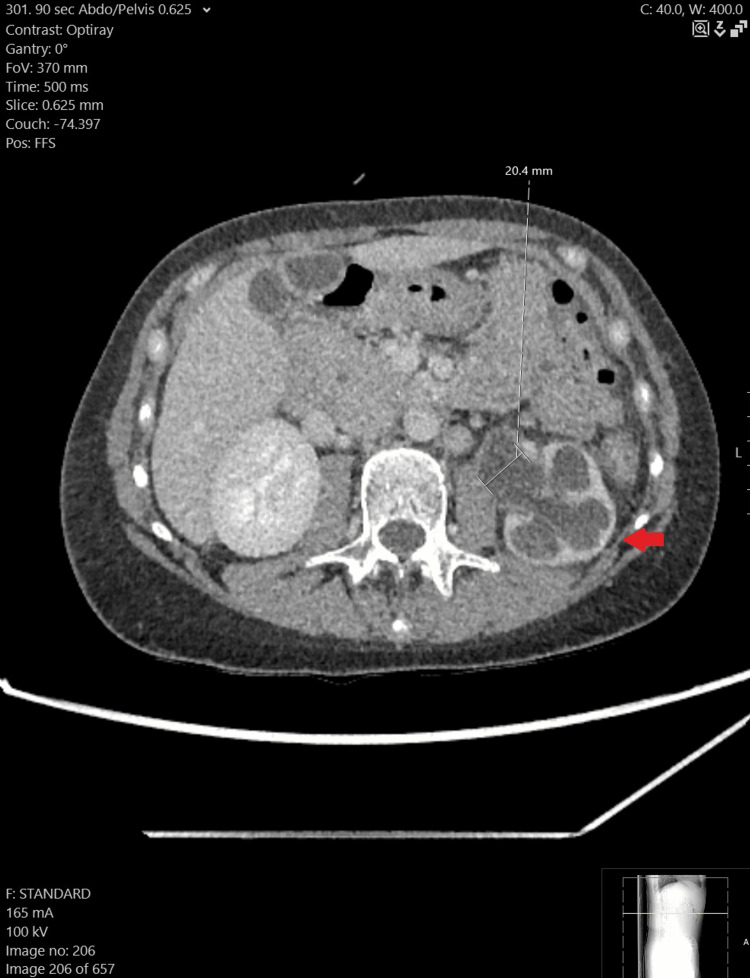
Axial image of the nephrographic phase of the CT urogram showing left-sided hydronephrosis (arrow)

**Figure 5:**
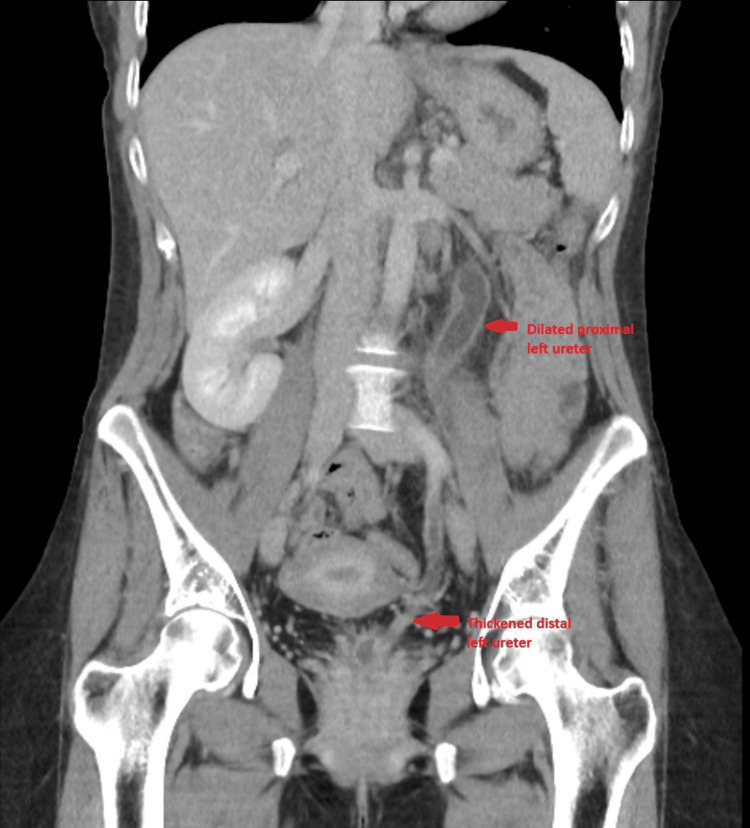
Coronal image of the nephrographic phase of the CT urogram showing a thickened left distal ureter and upstream dilatation (arrow)

Contrast leakage was shown in the urethra in keeping with incontinence (Figure 6), with no contrast leakage to the vagina to suggest vesicovaginal fistula.

**Figure 6:**
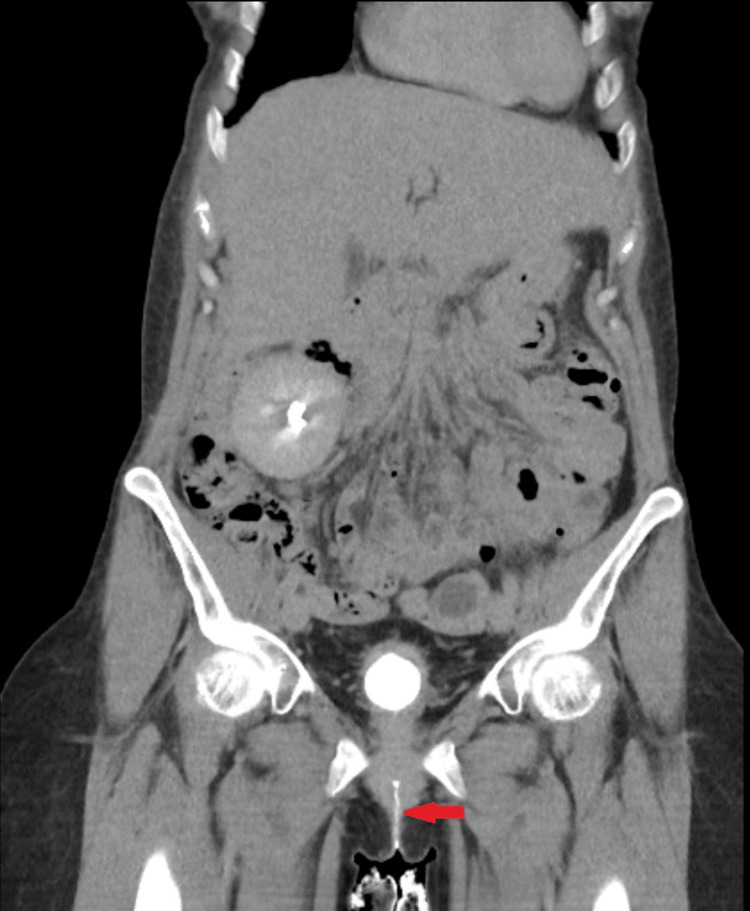
Coronal image of the CT urogram in the delayed phase showing contrast in the urethra (arrow)

In addition to imaging, she was investigated with a full urine report and culture, renal profile, serum inflammatory markers, blood white cell count, and liver function tests. Her urine culture and full report revealed positive for *Enterococcus *species, with a high white cell count suggestive of infection (Table 1). This likely represents a secondary urinary tract infection complicating the underlying urinary stasis, which drove the acute admission.

**Table 1:** Biochemical investigations, including blood and urine analyses on admission and at follow-up visits eGFR: estimated glomerular filtration rate

Biochemical investigations	Patient's value on admission	Follow-up visit in 2 weeks	Follow-up visit in 4 weeks	Normal range
Blood investigations				
WBC	14 x 10^9^	6.5 x 10^9^	8.1 x 10^9^	4-11 x 10^9^
Serum alanine aminotransferase (ALT)	318	235	79	10-50 U/L
Serum alkaline phosphatase (ALP)	300	249	216	30-130 U/L
Serum creatinine	65	60	69	65-119.3 micromol/L
eGFR	>90	>90	>90	
CRP	48	11	1	<10 mg/L
Urine analysis				
Urine WBC	20,801.5 x 10^6^/L			<30 x 10^6^/L
RBC	136.3 x 10^6^/L			<40 x 10^6^/L
Bacterial cells	1851.6 x 10^6^/L			<500 x 10^6^/L
*Enterococcus *species	Positive 10^8^ CFU/mL			

Her renal profile was normal despite the presence of moderate left-sided hydronephrosis. She was treated with antibiotics and reviewed in the urology clinic two weeks later. Follow-up blood investigations showed that the infection had resolved (Table 1).

Persistently high serum ALP and ALT levels were observed in two-week intervals following her admission, and ketamine-induced cholangiopathy was suspected. She was referred to the Gastroenterology Unit for further evaluation and workup.

Diagnosis of KIU was made depending on the characteristic imaging findings and the relevant clinical history. She was encouraged to stop ketamine use and referred to a specialised centre for further management. She is under local urology clinic follow-up while being followed at the specialised centre. She was also seen by the hospital substance abuse team and counselled.

## Discussion

KIU is a challenging urological condition with an increasing incidence over the last decade among the young population [7]. In a recent survey conducted in the UK, ketamine-abuse in people aged 16-24 years has increased from 1.9% in 2008/2009 to 2.9% in 2018/2019 [8]. Patients with KIU will commonly present with ‘K ‘(ketamine) cramps - severe abdominal and bladder cramping, such as pain, urinary incontinence, increased urinary frequency, urgency, and haematuria [2,7,8]. The symptoms may not occur acutely, and the effects are quantity- and duration-dependent [4]. The mechanism of action is not fully understood, but postulated that the direct toxicity of metabolised to the mucosa, leading to chronic inflammation [7]. A similar clinical presentation can occur with urinary tract calculi and infection. Therefore, detailed clinical history and biochemical investigations are important to establish a diagnosis in the acute clinical presentation.

The consensus guideline for the management of KIU by the British Association of Urological Surgeons recommends renal profile, liver profile, and renal tract ultrasound as baseline investigations in patients suspected of KIU [4]. Urinalysis and urine culture, together with renal and bladder ultrasound, are recommended as first-line investigations in patients aged 16-30 years presenting with storage lower urinary tract symptoms (LUTS) and pelvic pain [8].

Imaging features of the effects of chronic ketamine use in the urinary tract system are well documented in the literature. The urinary bladder is commonly involved. This is shown in the ultrasound and in CT imaging as a small volume with a thickened bladder wall [2]. A retrospective study done using CT urograms in Taipei Veterans General Hospital from 2006 to 2011, who presented with severe and intertractable LUTS following chronic ketamine abuse, showed that the majority of patients had diffuse bladder wall thickening and small volume bladder [9]. Our patient had a similar imaging appearance (Figure 1). The bladder volume can be calculated with ultrasound in pre and post-void status, allowing the calculation of residual volume. Urine stasis may predispose to infection, which our patient had.

CT urogram is the appropriate protocol used to delineate the extent of the disease, to exclude other causes of the symptoms, and to identify potential complications in patients with KIU. Multiphase CT urography comprises non-contrast, nephrographic, and delayed excretory phases, resulting in a higher radiation dose compared with the split-bolus technique [9]. Given that this condition commonly affects younger patients, the split-bolus technique is often preferred to minimise radiation exposure [9]. In addition to ketamine cystitis, the upper urinary tracts may be affected, resulting in ureteric wall thickening, which may be segmental, and dilatation of the ureters, including the pelvicalyceal systems, as demonstrated in our patient (Figures 3-4). Similar imaging appearances can also be seen in many other disease entities, namely, secondary to infection like tuberculosis (TB) and schistosomiasis, chronic renal tract inflammation due to nephrolithiasis, radiation induced cystitis, and urothelial carcinoma [2,10].

Urinary tract TB occurs as post-primary TB, and it is rare to have isolated ureters and bladder involvement [2]. A chest X-ray or CT chest is helpful to assess previous exposure to pulmonary TB. The diagnosis can be made with urine sampling for TB culture and bladder tissue sampling for histology [2]. Bladder schistosomiasis is a parasitic infection of the bladder. On imaging, a small contracted bladder with calcifications is the hallmark finding [11]. Vesico-ureteric reflux can occur, resulting in hydroureteronephrosis. Definitive diagnosis is made with urine microscopic analysis for schistosomiasis ova [11].

KIU is prevalent in the younger age group, and urothelial carcinoma is unlikely in the right clinical setting. Cystoscopy and biopsy are useful to establish the diagnosis in a doubtful case. Cross-sectional imaging will exclude the presence of urinary tract calculi. The patient's clinical background is helpful in identifying previous pelvic malignancy and radiotherapy when considering radiation cystitis.

It is important for the reporting radiologist to consider these differential diagnoses when interpreting the initial imaging findings. Accurate radiological interpretation also relies on the provision of adequate clinical history by the referring team. MR urography is also helpful for assessment of the ureters, but is not commonly used. Nuclear medicine studies, such as MAG-3, are helpful to assess the degree of ureteric obstruction and quantification of renal function [2].

Although the imaging features are suggestive of KIU, it is recommended to perform a cystoscopy with or without biopsy, as most of the patients will have visible haematuria [4]. Video-urodynamics can provide anatomical and functional information that is needed for accurate diagnosis, but this is poorly tolerated and can be reserved for those for whom urinary tract reconstruction might be considered [4]. Ureteric strictures and vesico-vaginal fistula are known complications of KIU [8].

The mainstay of treatment is cessation of ketamine use [2,4,7]. There are conservative and minimally invasive management options, such as botulinum injection for the bladder wall [2,10]. In cases of contracted bladder and upper tract involvement, early surgical intervention - such as partial cystectomy, enterocystoplasty, and ureteral reimplantation - can mitigate further deterioration and restore functionality [7].

Evaluation for cholangiopathy, nasal septal perforation in patients with snorting, and endocarditis in IV drug use are recognised associations with chronic ketamine use. Consideration of these entities, depending on the clinical presentation, is also important in overall patient management, highlighting the need for a multidisciplinary approach.

## Conclusions

Ketamine abuse is a growing medical issue impacting the quality of life. It is a burden to the health system. KIU is a well-established phenomenon, and typical imaging features are well documented. The characteristic imaging features include a small volume thick-walled bladder, ureteric wall thickening, and hydroureteronephrosis. The radiographic appearance overlaps with other inflammatory conditions in the urinary tract. CT urogram is the main imaging modality in which the delayed phase will demonstrate its complications. Urinary tract infection secondary to urine stasis may be a cause for acute clinical presentation without an established diagnosis of KIU. As the reporting radiologist, awareness of the condition is paramount to prevent delaying diagnosis. It is also important to elicit an adequate clinical history from the patient when the typical imaging and biochemical findings are present. A multidisciplinary approach is required in the management of these patients.
